# “Children of War”: Examining the Associations between War Exposure, Maternal PTSD, and Continuous Traumatic Stress on Israeli Children’s PTSD

**DOI:** 10.1007/s10802-025-01321-1

**Published:** 2025-04-24

**Authors:** Lilach Rachamim, Roy Aloni, Hila Mualem-Taylor, Oriana Glickman, Asaf Goodman, Nathaniel Laor

**Affiliations:** 1 Donald J. Cohen & Irving B. Harris Resilience Center, Oti Association, Tel-Aviv, Israel; 2 Department of Psychology, Ariel University, Kiryat HaMada 3, Ariel, 40700 Israel; 3 Tel Aviv Faculty of Medicine and Health Sciences, Tel Aviv University, Tel Aviv, Israel; 4 Child Study Center, Yale University, Conn, USA

**Keywords:** Continuous Traumatic Stress, Children, PTSD, Maternal PTSD, War, Risk Factors

## Abstract

Children in war-torn areas are highly susceptible to post-traumatic stress symptoms (PTSS), influenced by direct exposure to war and maternal PTSS. This risk is further heightened by continuous traumatic stress (CTS). However, the relationship between war exposure, maternal PTSS, CTS, and PTSS in children, particularly in different age groups, has not been extensively studied. The current study investigated Israeli children, with a specific focus on treatment-seeking preschoolers (ages 3–7) and school-aged children (ages 8–12). The sample included 220 dyads of children aged 3–12 and their parents, who were seeking treatment for children’s PTSS after the October 7th terrorist attack. They underwent a clinical assessment including sociodemographic information and filled out validated self-report and parent-report questionnaires assessing PTSS. Overall, 69% of preschoolers and 49.2% of school-aged children exhibited probable PTSD, along with 32.4% of their mothers. Maternal PTSS significantly predicted PTSS in preschoolers (*b* = 0.24, *SE* = 0.14, *p* <.01), whereas war exposure significantly predicted PTSS in school-aged children (*b* = 0.81, *SE* = 3.84, *p* <.05). The relationship between CTS condition and children’s PTSS was indirectly associated through maternal PTSS, solely among preschool children (*b* = 4.81, *SE* = 1.78, 95% CI [1.84, 8.69]). The study highlights early intervention’s need to target age-specific vulnerabilities to PTSS in children. It stresses the importance of enhancing parental skills and improving children’s resilience towards current and future traumas, particularly in conflict-affected areas. Healthcare services should provide trauma-focused treatment for parents and children to prevent exacerbating symptoms.

## Introduction

On 7 October 2023, Israel was attacked by Hamas, who invaded southern Israel, resulting in the loss of over 1,500 lives, many of which were civilians’. The “Swords of Iron” war ensued when the Israeli Defense Forces entered the Gaza Strip. Since the breakout of this armed conflict, thousands of Israeli families have been forced to evacuate their homes near the frontlines (Levkovich & Labels, 2024), while Palestinian families who lived in the north of the Gaza Strip were forced to evacuate to southern Gaza (Abudayya et al., 2023). As a result of these traumatic events, civilians on both sides of the military conflict have been facing a serious mental health crisis with hundreds of thousands of children being impacted by the exposure to the harsh realities of war (Levi-Belz et al., 2024).

Recent systematic reviews and meta-analyses have shown that children are particularly vulnerable to developing psychological symptoms in the aftermath of trauma and distress, with great attention to post-traumatic stress symptoms (PTSS) and a diagnosis of post-traumatic stress disorder (PTSD) as the primary psychopathology (Blackmore et al., 2020; De Haan et al., 2020; Rezayat et al., 2020; Woolgar et al., 2022). According to the Diagnostic and Statistical Manual of Mental Disorders (DSM-5), PTSD consists of four clusters of symptoms– intrusion, avoidance, negative alterations in cognitions and mood, and arousal and reactivity (American Psychiatric Association, 2013). A systematic review of child mental health in post-war situations found high levels of PTSD (47%), depression (43%), and anxiety (27%) among affected children (Attanayake et al., 2009). Specifically, studies in the Middle East (Dyregrov et al., 2002; Elbedour et al., 2007; Punamäki et al., 2015) and Africa (Betancourt et al., 2009; Schaal & Elbert, 2006) have shown exceptionally high prevalence rates of PTSD (58–80%) among war-affected children. Similar evidence on the impact of war trauma has been established for adults in various populations. Recently, for example, in Ukraine, 25.9-32.9% of adults met diagnostic requirements for PTSD, with exacerbation levels among Ukrainian internally displaced persons (IDPs)(39.4%), and Ukrainian refugees abroad (47.2-69.5%) (Aloni & Ben Ari, 2024; Karatzias et al., 2023; Lushchak et al., 2024). Most of these studies administrated self-report validated questionnaires (Aloni & Ben Ari, 2024; Punamäki et al., 2015), while few administrated diagnostic interviews (Schaal & Elbert, 2006).

The nature of the exposure to the October 7th attack and its’ aftermath makes the current study valuable for identifying possible factors affecting children’s PTSD in times of intensifying armed conflict. A body of research clearly demonstrated the relationship between the amount and intensity of traumatic experiences and the severity of PTSS and the importance of understanding PTSD in children against the backdrop of the nature and extent of their exposure to trauma (Kuterovac et al., 1994; Slone & Mann, 2016; Thabet et al., 2008). In addition to the considerable effects of exposure to trauma, research has recognized parental PTSD as a significant factor that influences the PTSS experienced by children. A meta-analysis of 32 studies confirmed a significant relationship between parent and child PTSS following various traumatic events (Morris et al., 2012), including war experiences (e.g., Kapel Lev-Ari et al., 2024; Thabet et al., 2008). Furthermore, parent PTSS in the acute period after the trauma was found to be associated with later child PTSS, but not vice versa (Silverstein et al., 2022). However, the magnitude of this association differs between studies and populations. A recent comprehensive survey of 1,775 parent-child dyads from 16 studies demonstrated small-to-moderate cross-sectional and longitudinal associations, indicating the possibility of additional factors influencing the relationship (Silverstein et al., 2022).

One of these potential factors may stem from repeatedly facing an ongoing and protracted threat, typically lasting several years. This situation is commonly referred to as Continuous Traumatic Stress (CTS) or Type III trauma exposure (Kira et al., 2013), where an individual is repeatedly exposed to traumatic events in their daily life (Straker, 1987). CTS is a common phenomenon worldwide and can result from various traumatic situations, such as living under conditions of constant urban violence (Roach, 2013) or being exposed to continuous rocket shelling (Greene et al., 2018), the latter of which was the focus of the current study. Since 2001, the southern region of Israel has been subjected to missile attacks, including the firing of rockets and shells at all hours of the day.

Living under conditions of ongoing threat involves constant uncertainty, as well as constant alertness and preparedness (Ruby, 2002). Recurrent experience of traumatic stress impairs people’s ability to maintain a stable routine and creates a sense of threat, vulnerability, anxiety, confusion, uncertainty, and helplessness (Zimbardo, 2002). Research has indeed documented elevated distress among individuals who experienced CTS situations, including anxiety, depression, somatization, and helplessness, as well as elevated PTSS (Hobfoll et al., 2006; Itzhaky et al., 2017; Nuttman-Shwartz & Shoval-Zuckerman, 2016), which are widely recognized as the most prevalent long-term psychological result of CTS (Bleich et al., 2003). Consequently, children residing in conflict-affected areas may face a heightened risk of developing PTSS compared to the general population (Attanayake et al., 2009; Feldman & Vengrober, 2011). Interestingly, it was documented during the COVID-19 pandemic that the vulnerability to emotional distress among citizens who live under CTS conditions is greater compared to citizens not under CTS (Lahav, 2020).

As mentioned, the empirical literature shows a significant relationship between parent and child PTSS. However, the strength of this association varies across different studies and populations. Notably, this association appears stronger in populations experiencing continuous threats than those exposed to threats for a limited duration (Pat-Horenczyk et al., 2013). Research involving various groups living under continuous stress, such as Palestinians in the Gaza Strip and Israelis residing near the Gaza border has indicated that a parent’s PTSS is linked to their child’s PTSS (Feldman & Vengrober, 2011; Thabet et al., 2008). Moreover, it has been suggested that parental PTSD may play an important role in the relationship between parents’ ongoing stressors, such as CTS in conflict zones, and the development of PTSD in children (Greene et al., 2018; Pat-Horenczyk et al., 2013; Straker, 1987). For instance, mothers living under CTS conditions exhibited increased maternal distress, which in turn affected the distress levels of their young children, especially amongst preschoolers (Feldman & Vengrober, 2011; Laor et al., 1996). Hoffman et al. (2020) found that maternal distress mediated the effects of maternal torture on child adjustment in refugee families. Similarly, a recent study by Tarabay and Golm (2024) indicated that parental war trauma indirectly influenced offspring psychopathology through increased parental psychopathology.

### The Present Study

To the best of our knowledge, the current literature has primarily focused on children aged 5 years and under or on adolescents, and there is a notable lack of comparisons of real-time reactions during times of armed conflict between populations living under CTS and those not affected by such stressors. The present study therefore had two main objectives. First, it sought to elucidate the experiences of children who have been exposed to war-related trauma, with a particular emphasis on those affected by the October 7th attack. Specifically, the study aimed to contribute to a broader comprehension of the prevalence of children’s PTSS and to identify key risk factors, including war exposure and maternal PTSS. Second, the study investigated whether living under CTS is an exacerbating factor in the mental well-being of children, and explored the role of maternal PTSS in that association. We hypothesized that: (a) Among war-affected children who sought treatment, there would be a high prevalence of PTSD; (b) Exposure to war traumatic events and higher maternal PTSS would each significantly predict children’s PTSS, even after controlling for sociodemographic factors; (c) Children residing in areas characterized by CTS would exhibit elevated PTSS compared to those who are not living under CTS, and (d) Maternal PTSS would show an indirect association between CTS condition and children’s PTSS.

## Methods

### Procedure

The research was conducted during the ongoing Iron Swards War from 7 November to 13 June 2024 at an online clinic specializing in treating traumatized children at Oti Association’s Cohen-Harris Resilience Center. The online clinic is recognized by both the Israeli Health Ministry and the Israeli National Insurance (INI). Following the events of October 7th, Israeli child trauma victims are eligible to receive psychological treatment at clinics recognized by the Israeli Health Ministry, with costs covered by INI. Information on contacting various clinics, including the current online clinic, was shared daily across multiple media platforms, including TV, radio, and social media.

In the first month following the 7 October attack, the clinic needed to make adjustments to its treatment program in response to the outbreak of war. This included altering some of the content of the treatment modules (e.g., removing visual elements related to captivity and tunnels), as well as revising the evaluation and assessment processes due to the significant increase in referrals to the clinic. After this period, the following methods were implemented. Each treatment request underwent two phases of evaluation. The first phase included a structured initial screening session by Israeli-trained, M.A. psychology students with a good grasp of the Hebrew language and under the supervision of an experienced, certified clinical psychologist. Each screening session was conducted with at least one of the parents to derive sociodemographic information (e.g. age, marital status, residence), verify accessibility for online treatment (a personal computer with connection to the internet, availability of parent to support the child during the treatment); obtain a brief description of the traumatic event by the parent, and gain answers to essential questions on the child’s reactions to the traumatic event (e.g. nightmares, avoidance, flashbacks). In this phase, the parent gave orally informed consent for the participation of the child.

In the second phase, patients meeting the inclusion criteria (see below) underwent a comprehensive 2-hour clinical assessment by an accredited psychologist, meeting with the parent separately and with both the parent and child together. The clinical assessment with the parents began with a structured interview that focused on specific questions about the traumatic event following the October 7th (e.g., did the child witness dead bodies or injured individuals? Who was with the child during the event?). The interview also collected background information on the family and the child (e.g. major life events, focusing on the child’s developmental and educational history, as well as previous traumatic events and psychopathology). Lastly, the parents completed a PTSD self-report questionnaire to evaluate the severity of their own PTSD symptoms. Afterwards, both the child and the parent met with the clinician for an evaluation of the child’s PTSD symptoms. This evaluation involved a clinical interview in which the clinician engaged the child in a discussion about each symptom, allowing for a detailed understanding of the child’s symptoms and filling out age-matched PTSD scales (see below). The study received approval from the Ariel University Research Ethics Committee (AU-SOC-RA-20220621).

### Participants

A total of 374 children and their parents completed the first phase of structured initial screening. Out of these, 243 individuals were invited for the second phase for comprehensive clinical assessment because they met the treatment inclusion criteria: (1) fluency in Hebrew; (2) having elevated levels of PTSD symptoms according to DSM-5 following 7 October traumatic events; (3) being at least one month away from the October 7th as a minimum period for determining PTSD according to criterion F in the DSM-5 (American Psychiatric Association, 2013); (4) being between 3 and 12 years of age; (5) having at least one parent who can support and accompany the child in the assessment and treatment, and with no other significant psychopathology. Based on these criteria, 131 children were excluded from the study: 26 families were lost to contact, and 17 preferred face-to-face treatment. The rest of the families were excluded due to conditions that would not have allowed them to support the child throughout the intervention: for example, parental mental disability (severe PTSD or grief over a spouse/child)(*n* = 25), lifetime diagnosis of Pervasive Developmental Disorder (*n* = 2), Intellectual Developmental Disorder (*n* = 1), not having PTSD symptoms (*n* = 18), children over the age of 13 (*n* = 2), and children who applied for treatment within the first month following the outbreak of war (*n* = 40). All excluded cases were provided with various treatment options to ensure they received appropriate care based on their circumstances.

Out of the 243 children who were invited to participate in the second phase of the comprehensive clinical assessment, 23 children did not complete the evaluation and were excluded from the final sample. The final sample comprised 220 children, 55.5% female, aged 3 to 12.3 years (*M* = 7.79, *SD* = 2.05). A total of 216 mothers (4 missing values) aged between 27 and 52 years (*M =* 39.41, *SD* = 5.16) participated in the study, with 195 (88.6%) being married and 25 (11.4%) unmarried (divorced, widowed). Additionally, 83.8% reported that they were employed. More than half of the sample (121, 55.0%) resided in southern Israel, near the Gaza border, having been under the threat of CTS prior to October 7th.

### Measures

All parents and children included in the final sample underwent two evaluation phases, as described above. The sociodemographic information and child history information were gathered during the interview with the parent, who filled out a self-report scale regarding his own PTSD symptomology. The clinical information about the children’s PTSD symptoms was gathered during the interview with both parent and child when the clinician filled out the scales during the meeting.

#### Continuous Traumatic Stress (CTS)

In the current study, the status of CTS was determined by inquiring about participants’ residing locations. Previous research indicates that citizens living in the Western Negev region, near the Gaza Strip border, experience ongoing stress due to the Israeli-Hamas conflict, which has included rocket threats for many years (see Greene et al., 2018 for a systematic review). Specifically, participants residing in areas within 30 km of the Gaza Strip have just 45 s to reach secure shelter, as outlined by the Home Front Command’s guidelines for entry times into protected areas (Bayer, 2024). This situation is documented under CTS conditions.

#### Exposure To War-Related Traumatic Events

Exposure to war-related traumatic events was assessed via a clinical interview that evaluated exposure to traumatic events relating to October 7th. After the clinical interview, the events were categorized based on two categories from the Life Event Checklist for DSM-5 (LEC-5), physical assault and assault with a weapon (Gray & Slagle, 2006). Each event was rated according to Schoenleber et al.’s (2018) updated grading procedures on a scale from 0 to 3 based on the threat level: 0 = no threatening event; 1 = slight possibility of threat; 2 = probable potential for threat; 3 = definite threat. For example, a missile landing near a house, causing the house to shake, led to the family staying in a bomb shelter for 3 to 4 days. This situation was rated as “3” for war exposure due to the imminent danger posed by the missile. In another case, children were left without their parents in central Israel during the outbreak of the war, in a location that lacked a bomb shelter. This was rated as “1” for war exposure, as it acknowledged the general threat of war without presenting any direct risk. Additionally, a child who spent 27 h straight in a bomb shelter, fearing that terrorists might enter and witnessing bullet-riddled cars and dead bodies on the way out, was rated as “3” for war exposure due to the prolonged and direct threat perceptions experienced. According to Schoenleber and colleagues (2018), children with a rating of “3” in at least one category were classified as having high exposure, while those with ratings of “0” to “2” were classified as having low exposure.

#### Child PTSD Symptoms

For preschoolers children between the ages of 3–7, we administered the Young Child PTSD Checklist (YCPC; Scheeringa, 2013) during the clinical interview conducted with the mother and child with an expert psychologist. The YCPC assesses PTSD symptoms in children based on DSM-5 criteria. It includes 23 items rated on a 5-point Likert scale, with 20 items for symptom combinations and six for functional impairment. Total scores range between 0 and 96, and the suggested cutoff for a probable diagnosis is 26. The YCPC demonstrated good psychometric properties, including high test-retest reliability (0.87) (Scheeringa & Haslett, 2010) and fair-to-good concurrent criterion validity (Scheeringa, 2020). It was chosen as the only measure available for preschoolers based on DSM-5 criteria, showing acceptable internal consistency (*α* = 0.89). (Scheeringa, 2013). A high Cronbach’s alpha (*α* = 0.87) was found in the current sample.

For children between the ages of 8–12, we administered the Child PTSD Symptom Scale for DSM-5 (CPSS-5; Foa et al., 2018). The CPSS-5 consists of 20 PTSD symptom items rated on a 5-point scale, ranging from 0 (not at all) to 4 (6 or more times a week/severe), assessing frequency and severity. Total scores range from 0 to 80, with a suggested cutoff of 31 for a probable PTSD diagnosis. The CPSS-5 has shown excellent internal consistency (*α* = 0.92) and good test-retest reliability (*r* =.80) (Foa et al., 2018). It has demonstrated good convergent and discriminant validity with the Multidimensional Anxiety Scale for Children (MASC) and Child Depression Inventory (CDI). In the current sample, a high internal consistency was found (*α* = 0.87).

#### Parent PTSD Symptoms

Parent PTSD symptoms were assessed by the Posttraumatic Diagnostic Scale (PDS-5; Foa et al., 2016). The PDS-5 assessed 20 items for each of the 20 DSM-5 PTSD symptoms, The 20 items are rated on a 5-point scale of frequency and severity ranging from 0 (‘not at all’) to 4 (‘6 or more times a week/severe’) in assessing PTSD symptoms according to the DSM-5. Based on these 20 items, a PDS total score can be calculated, and the proposed cutoff of is 28 for a probable PTSD diagnosis. The PDS–5 showed excellent internal consistency (*α* = 0.95) and test-retest reliability (*r* =.90)(Foa et al., 2016). A very high internal consistency was found in the current sample (*α* = 0.93).

### Analytic Procedure

The study included 220 children divided into two age groups. Preschoolers (ages 3 to 7, *n* = 100) and school-age children (ages 8 to 12, *n* = 120). Initially descriptive statistics regarding the socio-demographic data and the prevalence of war exposure were calculated. For comparisons between age groups, a Student’s t-test was conducted where normality assumptions were met. For variables that violated normality assumptions, the Mann-Whitney U test and Welch’s t-test were utilized. Fisher’s exact test was conducted for the categorical variables. Further analysis included means, standard deviations, and percentages which were calculated for PTSS measures for both children and parents.

In addition, two separate hierarchical linear regression analysis models were calculated, with the children’s PTSS as the outcome variable for each age group. The first step included demographic factors of age, gender, maternal age, and family status, while the second step involved exposure to war-related traumatic events. The final step included maternal PTSS. Furthermore, we conducted independent samples t-test to compared children and mothers affected by CTS and those unaffected by CTS in regards to PTSS levels. Finally, to test the hypothesized indirect association of maternal PTSS in the relationship between CTS and children’s PTSS, we conducted separate indirect associations analyses for preschoolers and school-age children using the PROCESS macro for SPSS (Model 4; Hayes, 2022). Given the cross-sectional nature of our data, we acknowledge that causal interpretations cannot be made, and we, therefore, focus on indirect associations between variables (Hayes, 2022). All statistical analyses were conducted using SPSS software (v. 25, SPSS, Chicago).

### Power Analysis

Power analysis was conducted for the main statistical analysis. For the hierarchical regression with six predictors, assuming a medium effect size (*f*^*2*^ =.15), *α* =.05, and desired power of.80, G*Power 3.1 indicated a minimum sample size of 98 participants was required. To analyze indirect effects, we examined the association between CTS and children’s PTSS through maternal PTSS. Based on previous meta-analytic findings showing small-to-medium associations between parental and child PTSS, the pathways to c’ small effect (0.14) and medium effect (0.39) for the a and b paths, we used Kenny MedPower [Computer software] (2017, February). With *α =* 0.05 and a desired power of 0.80, a minimum sample of 65 participants was deemed be necessary to detect a significance for the indirect path with the parameters specified for each outcome. A sample size of 130 participants was needed for the indirect analyses.

## Results

The two groups (preschoolers and school-age children) differed in gender (*X*^*2*^ (1, 220) = 8.11, *p* =.004), with a higher frequency of girls in the preschoolers group. Additionally, the mothers of preschool children were significantly younger (*M* = 38.18, *SD* = 5.20) compared to the mothers of school-age children (*M* = 40.43, *SD* = 4.82) (*U* = 1626.5, *p* =.005). No differences were found between the groups in other sociodemographic variables (parent employment, marital status, and CTS). For more information and comparisons between the two age groups, see Table 1.

**Table 1:** Sociodemographic characteristics

Variables	Preschoolers (*n* = 100)		School-age children (*n* = 120)	
	*n*	*%*	*n*	*%*
Children Gender				
Girls	45	45	77	64
Boys	55	55	43	36
Mother Employment				
Working	86	86	95	79
Not working	13	13	22	18
Missing data	1	1	3	3
Mother Marital Status				
Married	90	90	105	87
Divorced	10	10	11	9
Separate	0	0	2	2
Widow	0	0	1	1
Single	0	0	1	1
Children CTS ^a^	61	61	60	50

Note. CTS = Continuous Traumatic Stress status

^a^ Reflects the number and percentage of participants fitting to CTS condition

### Exposure To War-Related Trauma Events

According to updated grading procedures (Schoenleber et al., 2018), 24.0% of preschoolers and 16.7% of school-aged children were highly exposed to at least one definite threat related to war. The children experienced various traumatic events, with the most reported incidents being exposure to missile attack alarms (99.5%), direct missile impacts on their residence or nearby (33.6%), and terrorist infiltration and gunfire sounds (20.4%). The prevalence of children who were exposed to war-related traumatic events is presented in Table 2.

**Table 2:** Prevalence of exposure to major war-related traumatic events

Event	Preschoolers (*n* = 100)		School-age children (*n* = 120)	
	*n*	*%*	*n*	*%*
Missile alarm	100	100	119	99
Missile hits in their home/near them	38	38	36	30
Terrorist infiltration and Gunfire Sound	24	24	21	18
An extended stay in the safe room for more than 24 h	21	21	15	15
Harsh and explicit content in media	13	13	17	14
Witness severe damage to property during the war	14	14	11	9
Witness to people injured or bodies	7	7	4	3

### Prevalence and Associations of PTSD Among Children and Mothers

The prevalence of children meeting the DSM-5 criteria for probable PTSD was notably high. In terms of overall PTSD scores, 69.0% (69) of young children (ages 3–7) and 49.2% (59) of school-age children (ages 8–13) surpassed the clinical cut-off for probable PTSD. Additionally, 32.4% of parents (70) met the criteria for probable PTSD.Table 3 presents PTSS means and standard deviations according to total score and PTSD subscales (intrusions, arousal, avoidance, and change in cognition and mood).

**Table 3:** Ranges, means, and standard deviations of PTSD total scores and subscales in preschool children, school-age children, and their mothers

Measure	Preschool children (*n* = 100)			School-age children (*n* = 120)			Mothers (*n* = 216)		
	*Range*	*M*	*SD*	*Range*	*M*	*SD*	*Range*	*M*	*SD*
PTSD- Total Score	0–92	33.88	15.97	0–80	28.20	11.04	0–80	21.72	16.23
Intrusions	0–4	1.09	0.73	0–4	1.44	0.95	0–4	1.17	0.99
Increased arousal	0–4	2.06	1.0	0–4	1.88	0.99	0–4	1.22	0.86
Avoidance	0–4	1.19	0.83	0–4	2.10	1.26	0–4	1.28	1.18
Change in cognition and mood	N/A	N/A	N/A	0–4	1.09	0.79	0–4	0.86	0.81

*Note. M* = mean; *SD* = standard deviation; PTSD-total score = sum score; Intrusions, Increased arousal, Avoidance, Change in cognition and mood = average score. Range = possible range of the scale. N/A = not applicable for preschool children

Furthermore, as shown in Table 4, the correlations between children’s total PTSS scores and their subscales demonstrated a positive correlation with the mothers’ total PTSS scores and subscales. However, this pattern was observed only in preschool children, not school-aged children. Additionally, among preschool children, exposure to war was significantly and positively related to PTSS in both the mothers and the children. However, for school-age children, this association was only evident concerning the mothers’ PTSS, and no significant relation was found between war exposure and the children’s PTSS.

**Table 4:** Correlations for study variables

	1	2	3	4	5	6	7	8	9	10	11
1. War Exposure	—	0.08	0.03	0.18	0.08	-0.02	0.36***	0.29***	0.35***	0.30***	0.32***
2. Children’s Total score	0.30**	—	0.78***	0.53***	0.85***	0.78***	− 0.04	− 0.01	0.01	− 0.07	− 0.04
3. Children’s Intrusions	0.26**	0.79***	—	0.32**	0.53***	0.50***	− 0.07	0.01	− 0.01	− 0.13	− 0.07
4. Children’s Arousal	0.25*	0.80***	0.46***	—	0.32**	0.12	0.05	0.13	0.17	− 0.04	− 0.03
5. Children’s Avoidance	0.22*	0.83***	0.55***	0.54***	—	0.59***	− 0.10	− 0.10	− 0.05	− 0.09	− 0.12
6. Children’s Cognition and mood	N/A	N/A	N/A	N/A	N/A	—	0.03	− 0.01	− 0.05	0.06	0.09
7. Mother’s Total score	0.36***	0.41***	0.31**	0.34***	0.39***	− 0.04	—	0.82***	0.89***	0.91***	0.90***
8. Mother’s Intrusions	0.35***	0.47***	0.37***	0.35***	0.43***	0.12	0.89***	—	0.69***	0.72***	0.66***
9. Mother’s Arousal	0.29***	0.36***	0.25*	0.31**	0.36***	0.03	0.82***	0.69***	—	0.72***	0.74***
10. Mother’s Avoidance	0.30***	0.32**	0.25*	0.30**	0.27**	− 0.13	0.91***	0.72***	0.72***	—	0.75***
11. Mother’s Cognition and mood	0.32***	0.32***	0.24*	0.26**	0.35***	− 0.09	0.90***	0.74***	0.66***	0.75***	—

Note. Values above the diagonal represent school-age children, and values below the diagonal represent preschool children. N/A = not applicable for preschool children

*p <.05, **p <.01, ***p <.001

### Associations Between Sociodemographic Background, War Exposure, Maternal PTSS, and Children’s PTSS

Two hierarchical linear regression analyses were conducted to examine predictors of children’s PTSS in preschoolers and school-age children. Each model included three steps. The first step included demographics (gender, age, mother age, and family status), the second step added war exposure (high/low), and the final step included maternal PTSS (see Table 5).

For preschool-aged children, the first step was not significant (*F*(4, 51) = 0.17, *p* =.96). In the second step, adding war exposure, significantly increased the explained variance by 15.9% (*p* =.002), so war exposure was found to be significant (*β* = 1.01, *p* =.05). In the final step, we inserted maternal PTSS, which contributed an additional 13.7% to the explained variance (*p* =.002), resulting in a significant final model that explained 31.3% of the total variance.

For school-aged children, the first step was not significant (*F*(4, 64) = 0.47, *p* =.76). In the second step, adding war exposure significantly increased the explained variance by 6.7% (*p* =.029), so trauma exposure was found to be significant (β = 0.79, *p* =.05). In the final step, adding maternal PTSS was found to be not significant. However, war exposure continued to be significant in the final step (*β* = 0.81, *p* =.03). The final model explained 10.8% of the total variance.

**Table 5:** Hierarchical regressions for predictors of children’s PTSS

		Preschool children (*n* = 100)				School-age children (*n* = 120)			
Step 1		*B*	*SE B*	*Β*	*R* ^*2*^ *Δ*	*B*	*SE B*	*β*	*R* ^*2*^ *Δ*
Overall model		18.23			0.017				0.041
Gender		-3.29	4.07	− 0.22		3.30	2.63	0.32	
Age		0.02	2.02	0.00		0.23	0.77	0.04	
Mother age		0.23	0.39	0.08		0.06	0.26	0.03	
Family Status		-1.49	5.69	− 0.10		3.98	3.35	0.38	
**Step 2**									
Overall model					0.159^**^				0.067^*^
Gender		-2.50	3.77	− 0.17		2.77	2.56	0.27	
Age		0.75	1.88	0.05		− 0.06	0.76	− 0.01	
Mother age		0.11	0.36	0.04		0.06	0.25	0.03	
Family Status		1.34	5.33	0.09		4.25	3.26	0.41	
War Exposure		15.24*	4.72	1.01^*^		8.28	3.70	0.79^*^	
**Step 3**									
Overall model					0.137^**^				0.00
Gender		-2.65	3.48	− 0.18		2.83	2.60	0.27	
Age		− 0.16	1.75	− 0.01		− 0.08	0.78	− 0.01	
Mother age		0.53	0.35	0.19		0.06	0.25	0.03	
Family Status		5.90	5.11	0.39		4.14	3.36	0.40	
War Exposure		7.66	4.94	0.51		8.43*	3.84	0.81^*^	
Maternal PTSS		0.44**	0.14	0.46^**^		− 0.01	0.08	− 0.02	

*p <.05, **p <.01

### Continuous Traumatic Stress and PTSS of Mother and Child

To compare PTSS between mothers living under CTS conditions and those who were not, we conducted independent samples t-tests. The results demonstrated that mothers in the CTS condition had significantly higher PTSS scores (*M* = 25.72, *SD* = 16.51) compared to those who were not in the CTS condition (*M* = 16.81, *SD* = 14.52), with a moderate effect size (*t*(214) = -4.16, *p* <.001, *d* = − 0.57). However, no significant differences were found in PTSS scores between children living in CTS conditions and those who did not in both younger and older children’s groups.

Secondly, we examined the indirect association between the CTS condition and children’s PTSS through maternal PTSS symptoms. We conducted analyses using the PROCESS macro (Model 4) with 5000 bootstrap samples for each age group. For preschoolers, the CTS condition was significantly associated with higher maternal PTSS (*b* = 11.93, *SE* = 3.24, *p* <.001), and maternal PTSS was significantly associated with children’s PTSS (*b* = 0.40, *SE* = 0.10, *p* <.001). Although the direct association between CTS and children’s PTSS was not significant (*b* = -2.59, *SE* = 3.24, *p* =.43), bootstrap analysis revealed a significant indirect association through maternal PTSS (*b* = 4.81, *SE* = 1.78, 95% *CI* [1.84, 8.69]). The total association was not significant (*b* = 2.22, *SE* = 3.29, *p* =.50), with the model explaining 16.2% of the variance in preschooler’s PTSS (*R²* = 0.16, *p* <.001). For school-age children, the association between CTS and maternal PTSS was not significant (*b* = 5.75, *SE* = 2.97, *p* =.06), as well as maternal PTSS and children’s PTSS (*b* = 0.04, *SE* = 0.07, *p* =.52). The direct association between CTS and children’s PTSS was found not significant (*b* = -3.48, *SE* = 2.09, *p* =.099), as well as the indirect association (*b* = 0.24, *SE* = 0.50, 95% *CI* [-0.53, 1.50]). The model explained only 2.6% of the variance (*R²* = 0.26, *p* =.24).

**Fig. 1:**
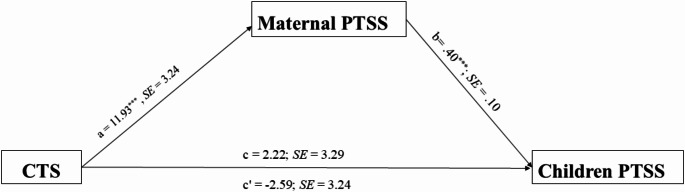
Indirect Effect of Maternal PTSS on the Association Between CTS and Preschooler's PTSS. Note. Values are unstandardized coefficients with standard error: CTS = Continous Traumatic Stress; PTSS = Posttraumatic Stress Symptoms. Indirect effect = 4.81, 95% CI [1.84, 8.69]. ***p <.001

**Fig. 2:**
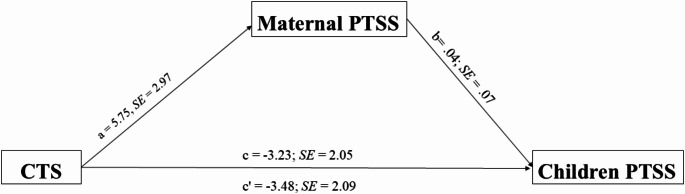
Indirect Effect of Maternal PTSS on the Association Between CTS and School-Age Children's PTSS. Note. Values are unstandardized coefficients with standard error: CTS = Continous Traumatic Stress; PTSS = Posttraumatic Stress Symptoms. Indirect effect =.24, 95% CI [-.53, 1.5]

These findings suggest an indirect association between the CTS condition and preschool children’s PTSS through maternal PTSS levels; however, this pattern was not observed in school-age children (see Figs. 1 and 2). In preschoolers, we observed an inconsistent pattern where the direct path coefficient was negative while the total association was positive, indicating that maternal PTSS reverses the direction of the relationship.For school-age children, the direct path coefficient was larger in magnitude than the total association because the small positive indirect path coefficient worked in the opposite direction. These patterns highlight different developmental pathways through which CTS relates to children’s PTSS (see Table 6).

**Table 6:** Indirect associations analysis for preschoolers and school age children

		B	SE	T	*P*	Lower CI	Upper CI
Preschool children	Total Effect	2.22	3.29	0.67	0.501	-4.31	8.74
	Direct Effect	-2.59	3.24	− 0.80	0.426	-9.03	3.84
	Indirect Effect	4.81	1.78	/	/	1.84	8.69
School-age children	Total Effect	-3.23	2.05	-1.58	0.117	-7.29	0.83
	Direct Effect	-3.48	2.09	-1.66	0.099	-7.61	0.66
	Indirect Effect	0.24	0.50	/	/	− 0.53	1.5

*Note. B* = unstandardized coefficient; *SE* = Standard Error; *t* = t-statistic value; *p* = p-value; *Lower CI* = Lower Limit of 95% Confidence Interval; *Upper CI* = Upper Level of 95% Confidence Interval

## Discussion

The study involved 220 mother-child dyads, where mothers sought treatment for their children aged 3 to 12 years due to elevated PTSS following the attacks on Israel on October 7th. The primary goal of this study was to contribute to a broader comprehension of the prevalence and risk factors linked to children’s PTSS, with a specific focus on war exposure and maternal PTSS. The second goal of this study was to investigate the relation of residing under CTS as an exacerbating factor in the mental well-being of children, as well as to analyze the indirect associations between CTS and children’s PTSS through maternal PTSS.

As hypothesized, we found a high prevalence of probable PTSD, with 69% of the preschool children (ages 3–7) and 49.2% of the school-age children (ages 8–12) meeting the criteria for PTSD. Additionally, 32.4% of parents met the criteria for probable PTSD. Our results are in line with the literature on the debilitating impact of war on children’s mental health (Halevi et al., 2016; Slone & Mann, 2016), and the prevalence of PTSD in our sample was comparable to that found in studies of children exposed to intense traumatic war experiences, such as those in Gaza Strip (El-Khodary et al., 2020; Thabet et al., 2008) and Southern Darfur (Morgos et al., 2007), where 70% of preschool children were found to have PTSD. As documented in the literature, trauma-exposed preschoolers are more prone to develop PTSD compared to school-age children (Martsenkovskyi et al., 2023). This indicates that the preschool years are a critical period of vulnerability due to the status of the development of linguistic and cognitive skills. Preschoolers lack advanced cognitive processes. Our results are congruent with studies indicating that young children lack advanced executive functions, such as self-regulation, mental flexibility, and inhibitory apparatuses, compared to school-aged children, exacerbating their risk profile for PTSD (Blair & Diamond, 2008; Feldman & Eidelman, 2009).

Our second hypothesis was partially confirmed according to age groups. Our results indicate that the event’s severity is related to school-age children’s PTSS but not preschool children. Differently, maternal PTSS was related to preschool children’s PTSS, but not to the event’s severity. Laor et al. (1996, 1997) posited that the “protective matrix”, which encompasses cultural, social, physical, familial, and personal adaptive mechanisms, may evolve with age. They suggest that preschool children rely more on their mothers’ symptomatology, with the mother’s stress-buffering function being a crucial component of this protective matrix. Over time, this idea has gained support in various trauma contexts, including single traumatic events such as the exposure to missile attacks during the Gulf War in Israel and the World Trade Centre attacks (DeVoe et al., 2011; Laor et al., 1997, 2001) Additionally it has been demonstrated in ongoing and repeated exposure to war-related traumatic events (Feldman & Vengrober, 2011; Pat-Horenczyk et al., 2013). It has been found that preschool children are more vulnerable to relational trauma within this protective matrix, especially when CTS increases maternal vulnerability, consequently worsening post-traumatic stress symptomology in the child (Pat-Horenczyk et al., 2013). Conversely, for school-age children, the role of the mother in PTSD seems to be less significant; instead, the exposure to traumatic events itself becomes a more critical factor, indicating that the protective matrix is dynamic and changes over time.

It is important to note that studies have shown the deleterious effect of war on the development of executive functions, including attentional biases to threat and the ability to inhibit response (Bar-Haim et al., 2010; Mueller et al., 2021). These have a decisive effect on children’s prosperity and their active and adaptive integration into society (Ainamani et al., 2017; Scharpf et al., 2022). Moreover, the maternal role in shaping the conditions that allow for normal development across the first years of life is a robust finding (e.g., Feldman & Eidelman, 2009). Additionally, Scharpf et al. (2022) demonstrated that both maternal-child and paternal-child relationship quality were positively associated with executive skills and inhibitory control in young refugee children but not in older children.

Our third hypothesis lay in the opportunity to examine the existing literature in the context of children’s reactions to a real-time war by examining the manifestation of PTSD within dyads residing in conflict-affected zones (CTS status) before the outbreak of the Israel-Hamas war, in juxtaposition with dyads situated in non-conflict areas. Our hypothesis that dyads living in conditions of CTS would exhibit higher levels of PTSS than those not living in CTS conditions was partially confirmed. Although we did not observe differences in PTSS among children in both age groups according to CTS condition, we did find that mothers living under continual threat displayed significantly higher levels of PTSS than mothers who were not exposed to continual threat.

Additionally, we hypothesized that maternal PTSS would indirectly associate CTS with children’s PTSS.Our hypothesis confirmed only among preschool children. The CTS condition appears to be a possible predisposing element for the emergence of PTSS in preschool children exposed to war trauma, contingent upon the simultaneous presence of maternal PTSS. The observed pattern of association between child traumatic stress (CTS) and preschoolers’ post-traumatic stress symptoms (PTSS) reveals an intriguing reversal in direction. Although neither the direct effect nor the total effect was statistically significant, they indicate opposing trends. This reversal occurs because the significant positive indirect association through maternal PTSS is strong enough to change the relationship between the variables (Zhao et al., 2010; Zou et al., 2020). Our findings are in line with previous studies showing the impact of cumulative traumatic stress on child-mother dyads in very young children, indicating their dependence on the maternal capacity to flexibly adjust to continuous environmental stressors (Feldman & Vengrober, 2011; Laor et al., 1996, 1997; Pat-Horenczyk et al., 2017, 2020).

This study has several limitations. Firstly, the data was gathered from a population seeking treatment in times of ongoing war, indicating that the high prevalence of probable PTSD identified in this study may be higher than that in the general population. For example, a nationwide study of Ukrainian children revealed that 17.5% of preschoolers and 12.6% of school-age children had PTSD (Martsenkovskyi et al., 2023). In the future, it would be advantageous to include more validated scales for exposure (El-Khodary et al., 2020) and investigate more representative samples, encompassing diverse populations residing in Israel, including Arab Israeli children. In addition, future studies should add a representation of paternal and not only maternal PTSD symptoms. The cross-sectional design of this study limited our ability to make causal inferences and forecast the long-term trajectory of children’s symptomatology (Lai et al., 2017). Although analysis of indirect associations in cross-sectional studies offers valuable insights into potential mechanisms (Hayes, 2022), it should be approached cautiously, highlighting the necessity for longitudinal studies that better identify potential mechanisms among the population living under CTS. To address this, we intend to examine the current population using a longitudinal study design, which will provide more reliable insights into the development of PTSD among children over time. This approach may also shed light on issues such as spontaneous recovery (Kolassa et al., 2010) and the effects of treatment. Finally, as recent studies revealed (Mueller et al., 2021; Scharpf et al., 2022), future studies should pay attention to neuropsychological aspects in the dynamic of PTSD among children and adolescents, with attention to executive function and other emotional-cognitive processes.

Our study has several important clinical implications. The results indicate that the event’s severity directly affected school-age children, not preschool children. However, maternal PTSS affected the preschool children, regardless of the event’s severity or ongoing traumatic stress. First and foremost, it is crucial to screen parents for PTSS, especially those with young children experiencing ongoing traumatic stress. Healthcare services should provide trauma-focused treatment for parents and children to prevent exacerbating symptoms. Additionally, providing treatment for those living under CTS can help strengthen coping mechanisms such as problem-solving and cognitive and behavioral skills to differentiate between real danger and trauma reminders (Cohen et al., 2011), ultimately building resilience (Pat-Horenczyk et al., 2017). These interventions can enhance parental competency and improve children’s ability to cope with current and future traumatic events, especially in war-torn regions.

## Data Availability

The dataset is available by request from the corresponding author (RA).
